# Supplementary figures and images for: GCMS profiling of bioactive phytocompounds from Curculigo orchiodes Gaertn. root extract and evaluation of antioxidant, and antidiabetic activities: A computational drug development approach
Source: PLoS One. 2025 Nov 5;20(11):e0335403. doi: 10.1371/journal.pone.0335403 (PMC12588482; doi:10.1371/journal.pone.0335403)

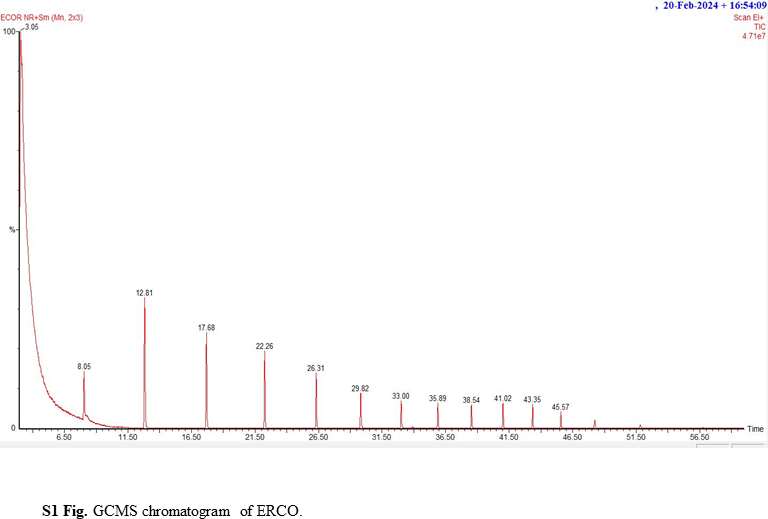

Supplement: S1 Fig — (TIF) [file pone.0335403.s001.tif]

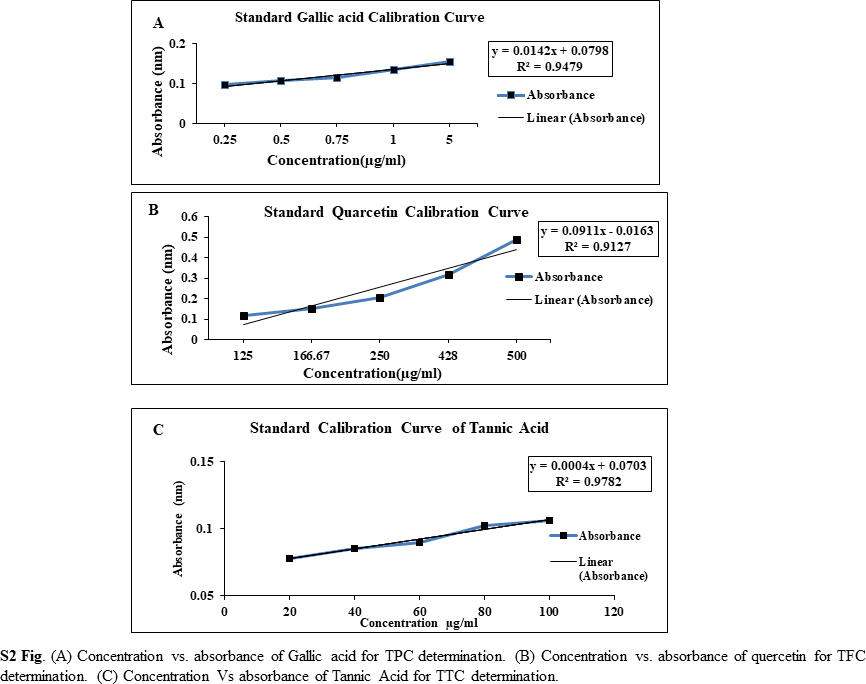

Supplement: S2 Fig — (B) Concentration vs. absorbance of quercetin for TFC determination. (C) Concentration Vs absorbance of Tannic Acid for TTC determination. (TIF) [file pone.0335403.s002.tif]
