# Supplementary material for: GCMS profiling of bioactive phytocompounds from Curculigo orchiodes Gaertn. root extract and evaluation of antioxidant, and antidiabetic activities: A computational drug development approach
Source: PLoS One. 2025 Nov 5;20(11):e0335403. doi: 10.1371/journal.pone.0335403 (PMC12588482; doi:10.1371/journal.pone.0335403)
Supplement: S1 Table — (DOCX) [file pone.0335403.s003.docx]

**S1 Table. GCMS Analysis of ERCO**

| **Serial No.** | **Retention**  **time (RT)** | **Name of the compounds** | **Molecular**  **weight** | **% Area** |
| --- | --- | --- | --- | --- |
| 1 | 3.05 | THIONYL CHLORIDE | 118 | 0.02 |
| 2 | 5.17 | ISOVALERIC ACID, 3-METHYLBUTYL-2 ESTER | 172 | 0.08 |
| 3 | 5.46 | L-METHIONINOL | 135 | 0.07 |
| 4 | 5.56 | 1,5-HEPTADIEN-4-OL, 3,3,6-TRIMETHYL- | 154 | 0.22 |
| 5 | 5.72 | 2-PROPANONE, 1,1,3,3-TETRACHLORO- | 194 | 0.19 |
| 6 | 6.09 | SUCCINIC ACID, DI(3,3-DIMETHYLBUT-2-YL) ESTER | 186 | 0.15 |
| 7 | 7.62 | DIFLUOROPHOSPHORIC ACID | 102 | 0.23 |
| 8 | 8.05 | NOREPINEPHRINE, (R)- | 169 | 8.18 |
| 9 | 8.20 | 2,2'-BI-1,3-OXATHIOLANE, 2-METHYL- | 192 | 2.86 |
| 10 | 8.62 | PROPANE, 1,1,3,3-TETRAMETHOXY | 162 | 0.32 |
| 11 | 12.81 | 1,2-EPOXY-3,4-DIHYDROXYCYCLOHEXANO[A]PYRENE | 302 | 22.28 |
| 12 | 17.68 | ETHYL GALLATE | 198 | 14.96 |
| 13 | 22.26 | 2,5-DIHYDROXYBENZOIC ACID | 154 | 11.20 |
| 14 | 26.31 | MERCAPTOACETIC ACID | 92 | 7.96 |
| 15 | 29.82 | HEXADECAMETHYL- | 533 | 29.82 |
| 16 | 33.00 | 3,4-DIHYDROXYPHENYLGLYCOL | 170 | 4.11 |
| 17 | 35.89 | 3,4-DIHYDROXYMANDELIC ACID | 184 | 3.39 |
| 18 | 38.54 | BORIC ACID | 62 | 3.27 |
| 19 | 41.02 | ANTHRACENE | 178 | 3.33 |
| 20 | 43.35 | MERCAPTOETHANOL | 78 | 3.10 |
| 21 | 45.57 | TETRADECAMETHYL- | 519 | 2.28 |
| 22 | 48.24 | FUMARYLACETOACETIC ACID | 200 | 1.28 |
| 23 | 51.82 | TARTRONIC ACID | 120 | 0.62 |
