# Supplementary material for: GCMS profiling of bioactive phytocompounds from Curculigo orchiodes Gaertn. root extract and evaluation of antioxidant, and antidiabetic activities: A computational drug development approach
Source: PLoS One. 2025 Nov 5;20(11):e0335403. doi: 10.1371/journal.pone.0335403 (PMC12588482; doi:10.1371/journal.pone.0335403)
Supplement: S2 Table — (DOCX) [file pone.0335403.s004.docx]

**S2 Table.** Docking Scores of 20 GC-MS-Identified Phytochemicals Compared with the Standard.

|  |  |  | **Anti-diabetic activity** | | | **Antioxidant activity** | | | |
| --- | --- | --- | --- | --- | --- | --- | --- | --- | --- |
| **SL.** | **Compounds** | **CID** | Alpha-amylase (1hny) | Alpha-glucosidase (3TOP) | SUR1 (5yw7) | Catalase (2CAG) | Peroxiredoxin 5 (1HD2) | Superoxide dismutase (1CB4) | Glutathione Peroxidase (2P31) |
| **1** | 1,2-EPOXY-3,4-DIHYDROXYCYCLOHEXANO[A]PYRENE_ | 41322 | -9.1 | -8.8 | -9.1 | -8.2 | -7.6 | -6.8 | -7 |
| **2** | 1,5-HEPTADIEN-4-OL, 3,3,6-TRIMETHYL | 100197 | -5.2 | -5.2 | -5.3 | -5.8 | -4.6 | -4.2 | -4.4 |
| **3** | 2,2'-BI-1,3-OXATHIOLANE, 2-METHYL | 568485 | -4.4 | -4.4 | -4.3 | -4.4 | -4.4 | -3.5 | -4.6 |
| **4** | 2,5-DIHYDROXYBENZOIC ACID | 3469 | -6.2 | -6.6 | -6.2 | -6.2 | -5.4 | -4.7 | -5.3 |
| **5** | 2-PROPANONE, 1,1,3,3-TETRACHLORO | 12436 | -3.9 | -4.9 | -4.1 | -4.6 | -4 | -3.4 | -3.7 |
| **6** | 3,4-DIHYDROXYMANDELIC ACID | 85782 | -5.9 | -6.9 | -6.3 | -6.8 | -5.6 | -4.8 | -5.6 |
| **7** | 3,4-DIHYDROXYPHENYLGLYCOL | 91528 | -5.6 | -6.7 | -6.2 | -5.8 | -5.4 | -4.6 | -5.6 |
| **8** | ANTHRACENE | 8418 | -7.2 | -9 | -7.8 | -9.9 | -5.9 | -5.4 | -5.8 |
| **9** | DIFLUOROPHOSPHORIC ACID | 61681 | -3.8 | -4.1 | -3.8 | -3.8 | -3.2 | -3.3 | -3.5 |
| **10** | ETHYL GALLATE | 13250 | -6.1 | -6.7 | -6.1 | -6.8 | -5.3 | -4.9 | -5.5 |
| **11** | FUMARYLACETOACETIC ACID | 5280398 | -5.6 | -5.5 | -5.4 | -6.2 | -5.3 | -4.8 | -5.3 |
| **12** | ISOVALERIC ACID, 3-METHYLBUTYL-2 ESTER | 58845665 | -5.1 | -5.3 | -5 | -4.7 | -4.4 | -3.6 | -4.2 |
| **13** | L-METHIONINOL | 2724404 | -3.8 | -4 | -3.9 | -4.2 | -3.6 | -3.3 | -3.3 |
| **14** | MERCAPTOACETIC ACID | 1133 | -3.2 | -3.4 | -3.2 | -3.7 | 3 | -2.8 | -3.1 |
| **15** | MERCAPTOETHANOL | 1567 | -2.6 | -2.7 | -2.8 | -3 | -2.3 | -2.2 | -2.6 |
| **16** | NOREPINEPHRINE, (R) | 439260 | -5.7 | -6.8 | -5.9 | -6.2 | -5.2 | -4.8 | -5.1 |
| **17** | PROPANE, 1,1,3,3-TETRAMETHOXY | 66019 | -4.1 | -4.7 | -4.1 | -3.9 | -3.7 | -3.9 | -4 |
| **18** | SUCCINIC ACID, DI(3,3-DIMETHYLBUT-2-YL) ESTER | 57171542 | -6 | -6.5 | -5.9 | -7.1 | -5 | -4.7 | -4.6 |
| **19** | ARTRONIC ACID | 45 | -5 | -5.3 | -4.6 | -5 | -4.4 | -4.7 | -4.3 |
| **20** | HIONYL CHLORIDE | 24386 | -2.7 | -3 | -3.1 | -3.1 | -2.5 | -2.4 | -2.5 |
| **21** | Glibenclamide (standard) | 3488 | -8.4 | -8.9 | -9 | × | × | × | × |
| **22** | Ascorbic acid (standard) | 54670067 | x | x | x | -5.5 | -5.2 | -4.9 | -5.4 |
| **23** | Acarbose (standard) | 41774 | -7.6 | -8.3 | - | × | × | × | × |
