# Supplementary material for: GCMS profiling of bioactive phytocompounds from Curculigo orchiodes Gaertn. root extract and evaluation of antioxidant, and antidiabetic activities: A computational drug development approach
Source: PLoS One. 2025 Nov 5;20(11):e0335403. doi: 10.1371/journal.pone.0335403 (PMC12588482; doi:10.1371/journal.pone.0335403)
Supplement: S3 Table — (DOCX) [file pone.0335403.s005.docx]

**S3 Table.** ADMET and drug-likeness properties analysis of two compounds.

| **Properties** | **Model** | **Compounds** | |
| --- | --- | --- | --- |
|  |  | **CID: 41322** | **CID: 8418** |
| Absorption | Intestinal absorption (human) | 96.075 | 95.506 |
|  | Skin Permeability | -2.747 | -2.307 |
|  | Caco2 permeability | 1.209 | 1.525 |
| Distribution | VDss (human) | -0.236 | 0.577 |
|  | Fraction unbound (human) | 0.04 | 0.074 |
|  | BBB permeability | 0.658 | 0.517 |
|  | CNS permeability | -1.839 | -1.18 |
| Metabolism | CYP2D6 substrate | No | No |
|  | CYP3A4 substrate | Yes | Yes |
|  | CYP2D6 inhibitor | No | No |
|  | CYP3A4 inhibitor | No | No |
| Excretion | Total Clearance | -0.05 | 0.132 |
| Toxicity | AMES toxicity | Yes | Yes |
|  | Hepatotoxicity | No | No |
|  | Skin Sensitization | No | Yes |
| Physicochemical | Molecular weight | 302.32 | 178.23 |
|  | Num. H-bond acceptors | 3 | 0 |
|  | Num. H-bond donors | 2 | 0 |
|  | Molar Refractivity | 88.87 | 61.45 |
| Lipophilicity | Consensus Log P | 2.82 | 4.02 |
| Water Solubility | Log S (ESOL) | -4.03 | -4.48 |
|  | Solubility Class | Moderately soluble | Moderately soluble |
| Drug-likeness | Lipinski violation | 0 | 1 |
|  | Bioavailability score | 0.55 | 0.55 |
| Medicinal Chemistry | PAINs | 0 | 0 |
