# Supplementary material for: GCMS profiling of bioactive phytocompounds from Curculigo orchiodes Gaertn. root extract and evaluation of antioxidant, and antidiabetic activities: A computational drug development approach
Source: PLoS One. 2025 Nov 5;20(11):e0335403. doi: 10.1371/journal.pone.0335403 (PMC12588482; doi:10.1371/journal.pone.0335403)
Supplement: Attachment — Submitted filename: Response to Reviewers.docx [file pone.0335403.s008.docx]

**Reviewer’s Comments and Author’s responses**

Reviewer#1

1. *The current abstract presents a concise summary of results but lacks the necessary depth and cohesion to effectively convey the study's significance. It is recommended that the authors thoroughly revise the abstract to ensure it is not only informative but also engaging, providing a clear and compelling narrative that aligns with the objectives of the research.*

**Author’s response:** The abstract has been thoroughly revised

2. *Given that traditional medicinal practices often employ aqueous extracts for plant-based remedies, the exclusive use of ethanolic extracts in this study warrants further justification. The authors should elaborate on the scientific rationale behind selecting ethanol as the primary solvent, addressing its advantages over aqueous extraction in terms of phytochemical yield, bioactive compound solubility, or other relevant factors.*

**Author’s response:** In response to the reviewer’s suggestion, we have now elaborated on this rationale in the introduction of the revised manuscript, clarifying the advantages of ethanol extraction over aqueous methods in the context of this study**.** Basically, the aqueous solvent facilitates the extraction of polar molecules more (due to the better solubilization), but in ethanol solvent, both the polar and moderately polar molecules become extracted, which rationalizes the extraction yield. Moreover, the aqueous solvent is difficult to evaporate by rotary evaporator and sometimes supports more microbial spoilage of the crude extract. Additionally, the alcohol-based extraction process is very widely accepted and utilized worldwide in the natural product research arena [1-3].

3. *The introduction, while comprehensive, could benefit from greater conciseness and sharper focus. Specifically, excessive details on diabetes and the enumeration of chemical compounds in Curculigo orchioides may detract from the core narrative. It is advisable to refine this section, retaining only the most pertinent information that contextualizes the study and underscores the plant's therapeutic relevance.*

**Author’s response:** The introduction section has been revised, and the irrelevant information has been removed.

4. *In the methodology detailing the extraction process, please specify the exact volume of 95% ethanol utilized for the maceration of 450 grams of powdered plant material. Elaborate comprehensively on the implications of this comment, addressing its relevance to extraction efficiency, solvent selection, or potential variations in phytochemical yield. A detailed response will enhance the reproducibility and scientific rigor of the study.*

**Author’s response:** We have added some points to the methodology section.

5. *The current analysis merely reports the qualitative presence or absence of phytochemicals. To strengthen the study’s analytical depth, it is recommended to include the quantitative yield (e.g., mg/g or percentage) of each identified phytochemical per gram of ERCO. Such data would provide valuable insights into the relative abundance of different phytochemical classes, facilitating a more nuanced interpretation of their pharmacological or biological significance.*

Author’s response: The study was conducted focusing on two pharmacological activities (i.e. Antioxidant and antidiabetic) on crude extracts. Consequently, in antioxidant tests, the targeted TPC, TFC, and TTC amounts have been quantified (Figure S2 in the supplementary file). Similarly, in GC-MS analysis, the identified compounds’ amount has been quantified by the peak area of individual spectra (Figure S1 in Supplementary file). Furthermore, due to the limitations of our laboratory facilities, more detailed quantification could not be performed for the rest of the compounds. But in future endeavours, we will definitely do purification followed by detailed quantification of bioactive constituents using HPLC or other appropriate methods.

6. To further corroborate the phytochemical findings, the authors may consider employing HPLC analysis for quantitative confirmation. Utilizing optimal detection wavelengths for each class of compound would enhance the specificity and reliability of the results, aligning the study with established analytical standards in phytochemical research.

**Author’s response:** In fact, this was one of our students' project works for academic degree, and due to time limitations and laboratory facilities constraints, the chromatographic purification and the HPLC analysis could not be performed. But we have a plan to perform the HPLC test on purified compounds in our next endeavour.

7. *The figure 3 contains special characters that seems to be representing statistical analysis yet no reference has been made in the text or in figure caption to what (*, **, ****, ####, ns) these mean. Please denote correctly.*

**Author’s response:** Amended

8. *While the in-vitro amylase and glucosidase assays provide preliminary insights, they alone are insufficient to comprehensively evaluate ERCO's anti-diabetic potential. Incorporating cell-based assays would strengthen the mechanistic understanding of its efficacy. Furthermore, correlating these findings with in-vivo studies would yield a more robust and translational assessment.*

**Author’s response:** We appreciate the reviewer’s thoughtful comment. We agree that in vitro assays alone are not sufficient for a comprehensive evaluation of anti-diabetic efficacy. However, we would like to clarify that *in vivo* anti-diabetic studies were indeed conducted using alloxan-induced diabetic Swiss albino mice, as detailed in the Materials and Methods and Results sections of the manuscript. These in vivo experiments demonstrated a significant reduction in blood glucose levels at both 100 mg/kg and 200 mg/kg body weight doses of ERCO, thereby supporting the translational relevance of our in vitro findings.

While cell-based mechanistic assays were beyond the current study's scope, we acknowledge their value and will consider them in future work to further elucidate the molecular pathways involved.

9. *The ADME-related tables should be consolidated into a supplementary file for improved readability. Instead of Table 3, a* *bioavailability radar plot summarizing the key pharmacokinetic properties of the selected compounds would offer a more visually intuitive representation.*

**Author’s response*:*** We have placed the ADME-related table into the supplementary file (New #Table S3) and added a bioavailability radar plot (New figure#5) according to the instructions.

10. *While the molecular dynamics simulations are technically sound, the section would benefit from conciseness. A more succinct and focused presentation of the data, eliminating redundant descriptions, would enhance clarity and impact.*

***Comments with respect to the organization of figures***

a. *Figure 1 appears to be non-essential for the main manuscript and may be better suited for inclusion in the supplementary materials, should the editors deem it necessary.*

**Author’s response*:*** The figure 1 has been added in supplementary file.

b. *Figure 2 lacks panel designations (A and B), yet the text refers to "Figure 2A" and "Figure 2B." For clarity, panel labels should be incorporated if distinct sections of the figure are being referenced. (In a figure, when we denote A and B to a certain figure, it generally refers to the panels represented in figures and not to the content of the individual figure).*

**Author’s response*:*** The description of the figure 2 has been revised (New# Figure 1) as per instruction.

c. *Figures 3 and 4 would benefit from consolidation into a single figure. Additionally, the presentation of amylase and glucosidase activities should precede the in vivo experimental results to maintain a logical flow.*

**Author’s response*:*** Figure 3 and 4 has been merged into a single figure (New # Figure 2). Necessary correction has been done.

d. *Figures 5, 6, 7 and 8 should be reorganized into a square or grid-like format (New figure #4) rather than a linear arrangement to enhance visual coherence and optimize space utilization.*

**Author’s response:** Figure 5-8 has been reorganized according to the instructions.

e. *Figures 9, 10, 11 and 12 could be compiled into a single composite figure for a more streamlined presentation. A recommended reference for organizing molecular dynamics simulation results is the article "In-silico evidence of ADAM metalloproteinase pathology in cancer signaling networks," which provides an effective model for such consolidation.*

**Author’s response:** Figure 9-12 has been reorganized (New figure #6) according to the instructions and as per suggested reference article.

**References:**

1. Atwi-Ghaddar S, Zerwette L, Destandau E, Lesellier E. Supercritical fluid extraction (SFE) of polar compounds from Camellia sinensis leaves: use of ethanol/water as a green polarity modifier. Molecules. 2023 Jul 18;28(14):5485.
2. Alamsjah F, Agustien A, Jannah M, Muqarramah M. Antimicrobial Potential of Ethanol Extract from Some Plants Against Staphylococcus aureus and Pseudomonas aeruginosa. Online Journal of Biological Sciences. 2024 24(3): 451-457.
3. Melo T, Figueiredo AR, da Costa E, Couto D, Silva J, Domingues MR, Domingues P. Ethanol extraction of polar lipids from Nannochloropsis oceanica for food, feed, and biotechnology applications evaluated using lipidomic approaches. Marine Drugs. 2021 Oct 21;19(11):593.

**Reviewer#2:**

***Comment****: The researchers have conducted an acute toxicity study, and the results have been included in the manuscript. However, since the crude extracts were administered to the mice, it would be beneficial to also incorporate chronic toxicity study findings. Additionally, including kidney and liver function test results would further strengthen the manuscript*.

**Author’s response:** We appreciate the reviewer’s safety concern of crude extract. Actually, In acute toxicity test, the crude extract was tested at higher doses (2000 mg/kg body weight) for short period of time (48 hours) and no signs of abnormality/mortality was observed. Additionally, here in ADMET analysis of in-silico study, the toxicity profile of the crude extract was analysed where the extract was also found non-toxic.

As we mentioned earlier, it was a student academic project and a preliminary study on crude extract foucsing two major pharmacological assements (antioxidant and antidiabetic) where chronic study took longer period of time at small doses. So from such hypothesis, it has been planned to run the project initially with the acute toxicity data and if the extarct show potential pharmacological and drug-like property in ADMET analysis, the further chronic toxcity will be done with pure fraction of this extract in further extension of this project in near future which will be submitted as another new article .
